# Activated FXR Inhibits Leptin Signaling and Counteracts Tumor-promoting Activities of Cancer-Associated Fibroblasts in Breast Malignancy

**DOI:** 10.1038/srep21782

**Published:** 2016-02-22

**Authors:** Cinzia Giordano, Ines Barone, Valentina Vircillo, Salvatore Panza, Rocco Malivindi, Luca Gelsomino, Michele Pellegrino, Vittoria Rago, Loredana Mauro, Marilena Lanzino, Maria Luisa Panno, Daniela Bonofiglio, Stefania Catalano, Sebastiano Andò

**Affiliations:** 1 Centro Sanitario, University of Calabria, Arcavacata di Rende, CS, Italy; 2 Department of Pharmacy, Health and Nutritional Sciences, University of Calabria, Arcavacata di Rende, CS, Italy

## Abstract

Cancer-associated fibroblasts (CAFs), the principal components of the tumor stroma, play a central role in cancer development and progression. As an important regulator of the crosstalk between breast cancer cells and CAFs, the cytokine leptin has been associated to breast carcinogenesis. The nuclear Farnesoid X Receptor-(FXR) seems to exert an oncosuppressive role in different tumors, including breast cancer. Herein, we demonstrated, for the first time, that the synthetic FXR agonist GW4064, inhibiting leptin signaling, affects the tumor-promoting activities of CAFs in breast malignancy. GW4064 inhibited growth, motility and invasiveness induced by leptin as well as by CAF-conditioned media in different breast cancer cell lines. These effects rely on the ability of activated FXR to increase the expression of the suppressor of the cytokine signaling 3 (SOCS3) leading to inhibition of leptin-activated signaling and downregulation of leptin-target genes. *In vivo* xenograft studies, using MCF-7 cells alone or co-injected with CAFs, showed that GW4064 administration markedly reduced tumor growth. Interestingly, GW4064-treated tumors exhibited decreased levels of leptin-regulated proteins along with a strong staining intensity for SOCS3. Thus, FXR ligands might represent an emerging potential anti-cancer therapy able to block the tumor supportive role of activated fibroblasts within the breast microenvironment.

Leptin is a multifunctional adipokine with several biological activities ranging from regulating food intake and energy metabolism to modulating many other processes, such as reproduction, lactation, haematopoiesis, immunity, cell differentiation and importantly carcinogenesis^1^,^2^. A growing body of evidence indicates a crucial role of leptin in the pathogenesis of breast cancer. Particularly, it has been extensively demonstrated that this adipokine is an important pro-inflammatory, proangiogenic, pro-invasive and mitogenic factor^1^,^2^,^3^, whose actions are strengthened through interaction with other different signaling molecules such as estrogens, growth factors and inflammatory cytokines^4^,^5^,^6^,^7^.

Leptin may act via endocrine, paracrine, and autocrine manner in breast cancer^8^. Indeed, in addition to the adipose tissue that represents the main source of leptin, normal and malignant breast tissue also secrete this adipokine^9^. Leptin and its receptor (ObR) are overexpressed in breast cancer compared with non-transformed mammary gland and benign mammary tumors and both molecules positively correlated with poor prognosis in primary breast carcinoma^10^,^11^. Moreover, we recently demonstrated that leptin is also secreted by a subpopulation of fibroblasts, known as cancer-associated fibroblasts (CAFs), within the tumor microenvironment, and that CAFs-secreted leptin promotes proliferation, migration, and invasiveness of breast cancer cells^12^.

Since Paget’s “Seed and Soil” hypothesis^13^ on distribution of secondary growths in breast cancer, it took more than 100 years to definitively demonstrate that the phenotype of malignant cells is strictly dependent on heterotypic signals coming from stromal cells in the surrounding microenvironment. Indeed, stromal cells influence tumor invasiveness and malignancy, whereas at the onset and during breast cancer progression, the microenvironment is reorganized by cancer cells. In the almost all solid tumor microenvironment, CAFs are present in aberrantly high numbers and are distinct from normal fibroblasts, involved in the healthy tissue homeostasis^14^. Particularly, in breast malignancies, CAFs exert a pivotal role in tumor onset and progression through multiple mechanisms, such as affecting estradiol levels, secreting increasing levels of growth factors, chemokines, cytokines and matrix metalloproteinases (MMPs), inducing epigenetic changes, epithelial to mesenchymal transition (EMT) and stemness. More importantly, CAFs not only induce mammary carcinogenesis but also promote therapeutic resistance, which contributes to breast cancer progression and poor prognosis. Thus, targeting specific CAFs-secreted factors, such as leptin, may provide more effective treatment options to achieve therapeutic benefits in breast cancer patients.

The farnesoid X receptor (FXR) is an adopted member of the metabolic nuclear receptor superfamily, mainly expressed in the liver and in the gastrointestinal tract, where it regulates expression of genes involved in bile acids, cholesterol and triglyceride metabolism^15^,^16^,^17^. Recent findings extend its function in several nonenterohepatic tissues, including its control in regulating cell growth and carcinogenesis. Indeed, separate studies have established both positive and negative correlations between FXR expression and cancer^18^,^19^,^20^,^21^,^22^,^23^,^24^,^25^,^26^. Particularly, in breast cancer cell lines FXR agonists inhibit aromatase expression reducing local estrogens production and induce apoptosis^24^, whereas other authors have reported that FXR activation stimulates breast cancer cell proliferation^27^. Besides, we have shown that activated FXR decreases tamoxifen-resistant breast cancer cell growth reducing the membrane tyrosine kinase receptor HER2 expression and signaling^28^. It has also been demonstrated that FXR activation by natural and synthetic ligands represses the expression of inflammatory cytokines and chemokines^29^,^30^. Thus, FXR can be considered more than a metabolic regulator, and FXR ligands may represent an important research issue to provide an alternative therapeutic strategy for the treatment of breast cancer.

In this study, we investigated, using both *in vitro* and *in vivo* experimental models, the ability of the synthetic FXR agonist GW4064 to interfere with cancer-promoting activities of CAFs focusing on the possible opposing role of activated FXR on leptin-induced breast tumor growth and progression.

## Results

### Activated FXR Inhibits Leptin-Induced Growth and Motility in Breast Cancer Cells

**Figure 1:**
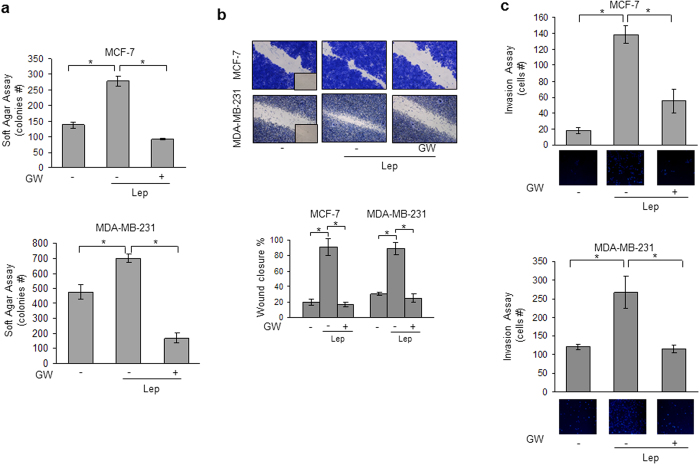
Activated FXR inhibits leptin-induced growth and motility in MCF-7 and MDA-MB-231 breast cancer cells. (**a**) Soft agar growth assays in MCF-7 and MDA-MB-231 cells treated with Leptin (Lep, 500 ng/ml) in the presence or absence of GW4064 (6 μM). After 14 days of growth colonies >50 μm diameter were counted. (**b**) Wound-healing assays in MCF-7 and MDA-MB-231 cells treated with Lep in the presence or absence of GW4064. Fields were photographed immediately after wounding (inset, time 0) and 24 or 12 hours later for MCF-7 and MDA-MB-231 cells, respectively. *Upper panel*, representative images from each condition are shown. *Lower panel*, the histograms represent the relative percentage of wound closure calculated by image analysis using Scion Image software. **(c)** Matrigel invasion assays in MCF-7 and MDA-MB-231 cells treated with Lep in the presence or absence of GW4064. The migrated cells were 4′,6-Diamidino-2-phenylindole (DAPI)-stained, counted and images were captured at 10X magnification. Typical well for each condition is shown. The values represent the mean ± SD of three different experiments, each performed in triplicate. *p < 0.05.

Leptin, acting in an autocrine, endocrine and paracrine manner, influences many aspects of breast tumorigenesis from initiation and primary tumor growth to metastatic progression. Thus, our first aim was to investigate the role of activated FXR on leptin-induced cell proliferation and motility using as experimental models poorly invasive/low metastasizing MCF-7 estrogen receptor (ER) α positive and highly invasive and metastatic MDA-MB-231 (ERα-negative) human breast cancer cells. Both MCF-7 and MDA-MB-231 cells were treated with leptin (500 ng/ml) with or without GW4064 (6μM), a synthetic FXR agonist, and growth was evaluated by anchorage-independent soft agar assays which closely mimic some *in vivo* biologic features of tumors. Leptin exposure increased colony numbers in both cell lines and this effect was completely reversed by GW4064 treatment (Fig. 1a). We then examined the ability of the FXR ligand to affect leptin-induced breast cancer cell movement in wound-healing scratch assays. Leptin-treated MCF-7 and MDA-MB-231 cells moved the farthest in either direction to close the gap compared to untreated cells, whereas GW4064 treatment was able to significantly inhibit leptin-induced migration (Fig. 1b). Moreover, we also tested the effect of GW4064 in counteracting leptin-induced capacity of MCF-7 and MDA-MB-231 cells to invade an artificial basement membrane Matrigel in invasion assays. As showed in Fig. 1c, leptin-increased invasion of breast cancer cells was completely abrogated by GW4064 treatment. The ability of the FXR ligand to block leptin actions was also reproduced in another ERα-negative breast cancer cell line, SKBR3 (Supplementary Fig. S1a and S1b online). These results clearly show that FXR activation affects leptin stimulatory effects on growth, motility and invasiveness in different breast cancer cell models.

### GW4064 Reduces Leptin Signaling Pathway Activation and Leptin Target Gene Expression in Breast Cancer Cells

**Figure 2:**
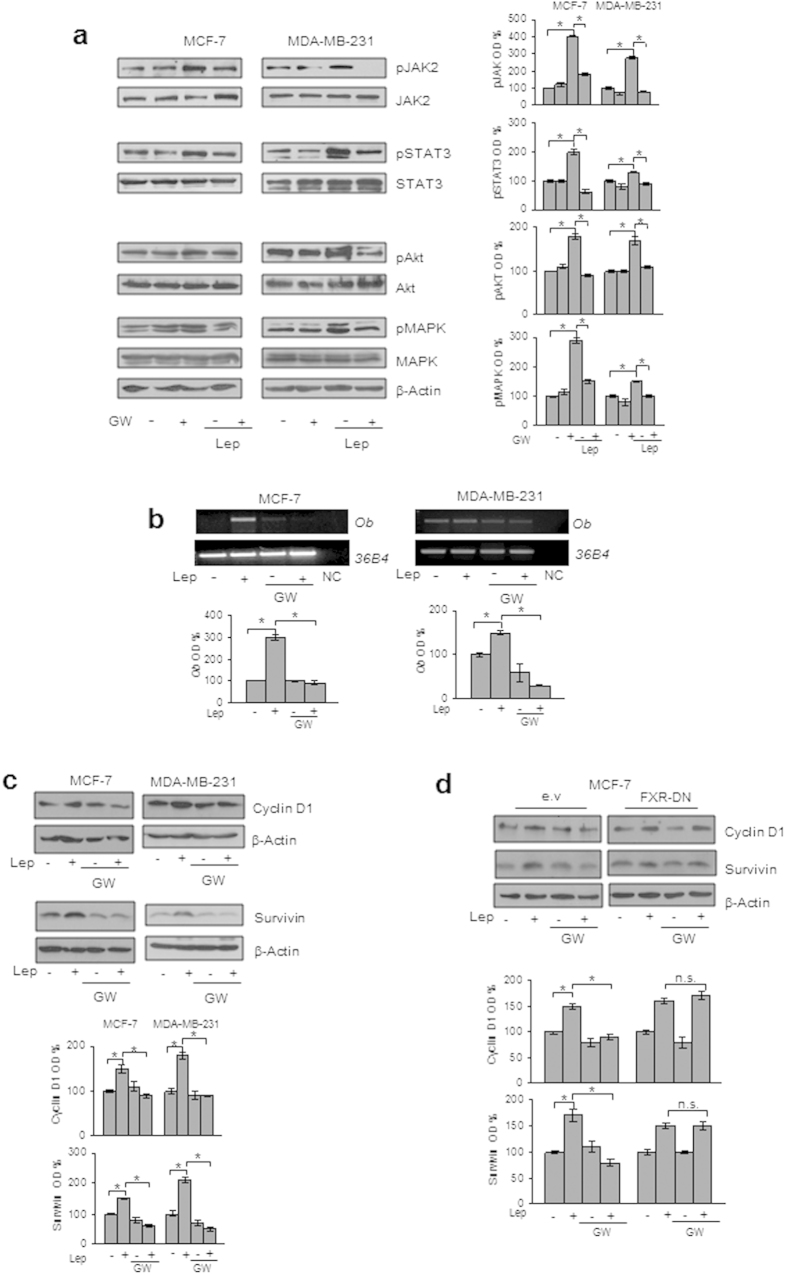
Effects of GW4064 on leptin signaling and its target genes in breast cancer cells. (**a**) MCF-7 and MDA-MB-231 cells were pretreated for 12 h with GW4064 and then treated or not for 10 min with Lep. Levels of phosphorylated (p) JAK2, STAT3, Akt, and MAPK, and total non-phosphorylated proteins were evaluated in cellular extracts by immunoblot analysis. β-Actin was used as loading control. (**b**) Total RNA was extracted from MCF-7 and MDA-MB-231 cells, pretreated for 12 h with GW4064 and then treated or not for 24 h with Lep, reverse transcribed and cDNAs were subjected to PCR using primers specific for *Ob* or *36B4* (internal control). NC: negative control. (**c**) Immunoblot analysis for Cyclin D1 and Survivin expression in MCF-7 and MDA-MB-231 cells pretreated for 12 h with GW4064 and then treated or not for 24 h with Lep. **(d)** MCF-7 cells were transiently transfected with either empty vector (e.v.) or FXR dominant negative plasmid (FXR-DN), pretreated for 12 h with GW4064 and then treated or not for 24 h with Lep. Cyclin D1 and Survivin expression levels were evaluated by immunoblotting. β-Actin was used as loading control. The histograms represent the mean ± SD of three separate experiments in which band intensities were evaluated in terms of optical density arbitrary units (OD) and expressed as percentage of vehicle-treated samples which were assumed to be 100%. n.s. = non significant, *p < 0.05.

Leptin exerts its biologic function through binding to its specific membrane receptor (ObR) able to activate multiple downstream signaling pathways^31^,^32^. First, we evaluated whether GW4064 treatment may affect the expression of leptin receptor (ObR) in breast cancer cells. As reported in Supplementary Fig. S2 online, we did not observe any significant changes in ObR mRNA levels after treatment with GW4064 for 12 and 24 h. Then, immunoblot analysis was performed to evaluate the phosphorylation levels of the major leptin downstream signaling molecules in cells pretreated or not with GW4064 for 12 h and then subjected to short-term stimulation with leptin. As expected, in both MCF-7 and MDA-MB-231 cells, leptin treatment resulted in increased phosphorylation levels of JAK2, STAT3, AKT and MAPK compared to untreated cells, whereas pretreatment with GW4064 abrogated the leptin activation of these signaling pathways (Fig. 2a). Next, we evaluated the impact of activated FXR on the expression of well-known leptin target genes, such as *Ob*, *Cyclin D1* and *Survivin*^33^,^34^,^35^,^36^,^37^,^38^. We observed that exposure to GW4064 significantly reduced leptin induction on *ObR* mRNA levels (Fig. 2b) as well as leptin-mediated upregulation of Cyclin D1 and Survivin protein content (Fig. 2c) in both MCF-7 and MDA-MB-231 cells. These effects were completely reversed in cells transiently transfected with a dominant negative FXR (FXR-DN) plasmid, supporting the direct involvement of this nuclear receptor in affecting leptin signaling in breast cancer cells (Fig. 2d). The negative regulatory role exerted by GW4064 on leptin-target genes was also reproduced in SKBR3 cells (Supplementary Fig. S1c and S1d online).

**Figure 3:**
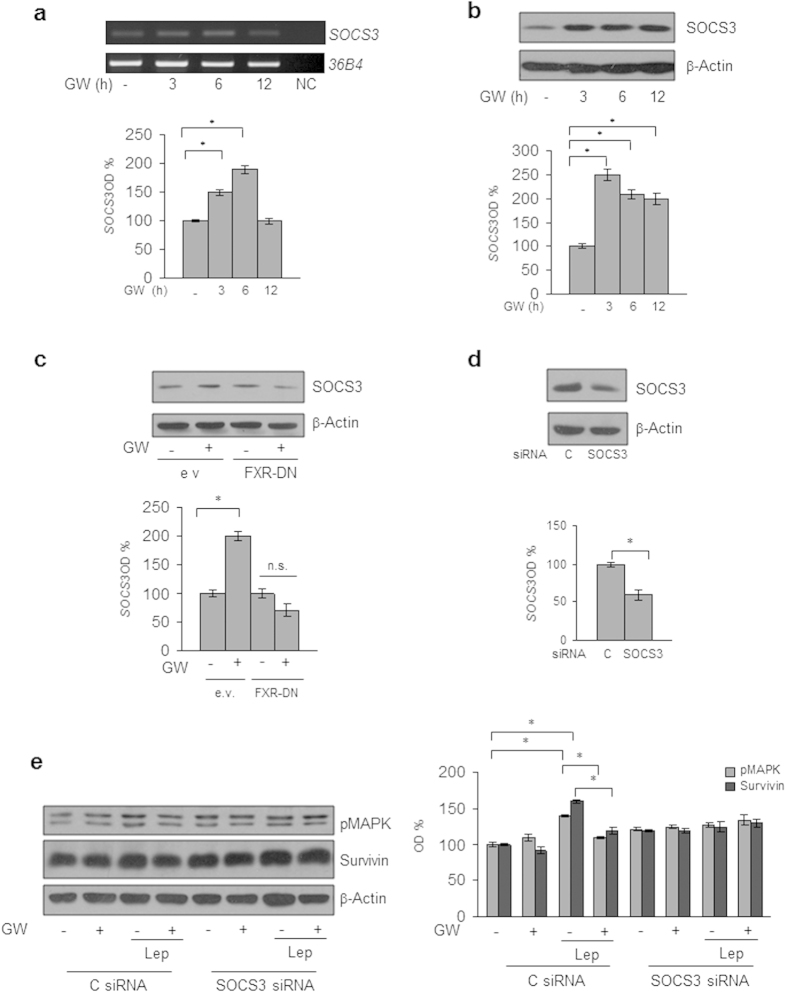
SOCS3 mediates GW4064 effects on leptin signalling molecules in MCF-7 breast cancer cells. (**a**) Total RNA was extracted from MCF-7 cells treated with GW4064 as indicated, reverse transcribed and cDNAs were subjected to PCR using primers specific for *SOCS3* or *36B4* (internal control). NC: negative control. Immunoblotting analysis for SOCS3 expression in total protein extracts from MCF-7 cells treated with GW4064 as indicated (**b**) or from MCF-7 cells transiently transfected with either empty vector (e.v.) or FXR dominant negative plasmid (FXR-DN) and treated with GW4064 (**c**) or from MCF-7 cells transiently transfected with either a control siRNA (C siRNA) or a siRNA targeted for the human SOCS3 RNA sequence (SOCS3 siRNA) (**d**). (**e**) Levels of phosphorylated (p) MAPK and Survivin were evaluated in protein extracts from MCF-7 cells transfected and treated as indicated by immunoblot analysis. β-Actin was used a loading control. The histograms represent the mean ± SD of three separate experiments in which band intensities were evaluated in terms of optical density arbitrary units (OD) and expressed as percentage of vehicle-treated samples which were assumed to be 100%. n.s. = non significant; *p < 0.05.

The suppressor of cytokine signaling 3 (SOCS3), the negative feedback regulator of leptin receptor signaling pathway^39^,^40^, has been recently identified as a direct FXR target gene^41^,^42^. Thus, to gain insights into the molecular mechanism underlying the inhibitory role of activated FXR on leptin transductional pathways, we conducted time-course studies to examine the effects of the FXR ligand on the expression of SOCS3 in MCF-7 cells. Treatment with GW4064 was able to increase the cellular content of SOCS3 at both mRNA and protein levels (Fig. 3a,b). The direct involvement of activated FXR in the regulation of SOCS3 expression in breast cancer cells was ascertained by evaluating SOCS3 content in the presence of FXR-DN plasmid. As shown in Fig. 3c, the expression of the FXR-DN completely abrogated the GW4064-induced SOCS3 levels. Finally, the functional relevance of SOCS3 in mediating GW4064 effects was confirmed by knocking-down its expression in MCF-7 cells (Fig. 3d). SOCS3 gene silencing abolished the inhibition exerted by GW4064 on leptin-induced effects on downstream signalling molecules, such as MAPK and Survivin (Fig. 3e). Taken together, these data demonstrate that modulation of SOCS3 expression may represent a mechanism through which FXR activation could affect leptin activity on breast cancer.

### GW4064 Reverses CAF-Induced Breast Cancer Cell Growth and Motility

**Figure 4:**
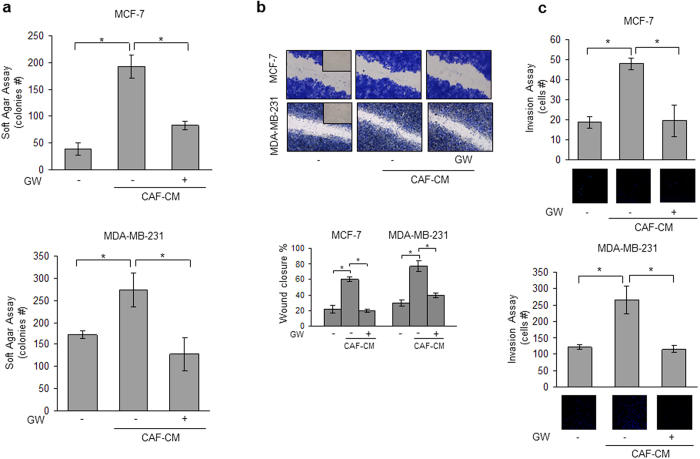
GW4064 effects on CAF conditioned media-induced growth and motility in breast cancer cells. (**a**) Soft agar growth assays in MCF-7 and MDA-MB 231 cells treated with CAF-derived conditioned media (CAF-CM) in the presence or absence of GW4064. (**b**) Wound-healing assays in cells treated as indicated. Fields were photographed immediately after wounding (inset, time 0) and 24 or 12 hours later for MCF-7 and MDA-MB-231 cells, respectively. *Upper panel*, representative images from each condition are shown. *Lower panel*, the histograms represent the relative percentage of wound closure calculated by image analysis using Scion Image software. (**c**) Matrigel invasion assays in cells treated as indicated. The migrated cells were DAPI-stained, counted and images were captured at 10X magnification. Typical well for each condition is shown. The data represent the mean ± SD of three independent experiments, each performed in triplicate. *p < 0.05.

Recently, we have identified leptin as one of the most important molecular player that mediates CAF effects in influencing tumor cell behavior. Indeed, we have shown that leptin immunodepletion from CAF-derived conditioned media (CAF-CM) substantially reduced the growth- and migration-promoting activities of CAFs on breast cancer cells^12^. On the basis of these data and since the activated fibroblasts are the principal cellular components in the breast stromal compartment, we investigated the ability of GW4064 to interfere with tumor microenvironment pressure. To this aim, CAFs were isolated from biopsies of four primary breast tumors and were used in co-culture systems. Isolated CAFs typically displayed the basic fibroblast characteristics with long and spindle-shaped morphology and highly expressed alpha-smooth muscle actin (α-SMA) and fibroblast activation protein (FAP) (Supplementary Fig. S3 online). Moreover, ELISA measurement in conditioned media from CAFs confirmed leptin secretion (2, 6 ± 0, 21 ng/mg protein). Both MCF-7 and MDA-MB-231 cells were incubated with CAF-CM and growth was evaluated by anchorage-independent soft agar assays. CAF-CM significantly enhanced colony numbers in both cell lines and treatment with GW4064 effectively counteracted the CAF-elicited increase in tumor cell growth (Fig. 4a). Then, we investigated the ability of the FXR ligand to inhibit the effects of conditioned media from CAFs on cell migration and invasion in MCF-7 and MDA-MB-231 cells. As shown in Fig. 4b, CAF-CM significantly accelerated closure of the cell-cleared area in both cell lines compared to control media, and GW4064 treatment was able to reverse these effects. Moreover, CAF-CM stimulation also increased the number of invaded cells in both cell lines and as expected treatment with GW4064 resulted in a clear reduction of cell invasion induced by CAF-CM (Fig. 4c). The inhibitory effects exerted by GW4064 on breast cancer cell proliferation and motility mediated by CAF-CM were also confirmed in SKBR3 breast cancer cells (Supplementary Fig. S1e and S1f online).

### The FXR ligand GW4064 Inhibits Tumor Growth in MCF-7/CAF xenografts

**Figure 5:**
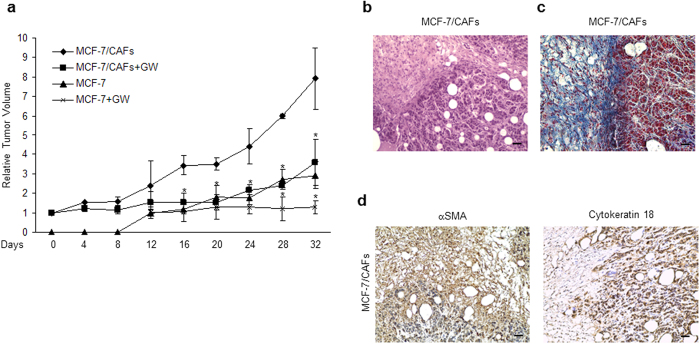
Impact of GW4064 treatment on tumor growth of MCF-7 and MCF-7/CAF xenografts. (**a**) MCF-7 cells were injected alone or coinjected with CAFs (MCF-7/CAFs) subcutaneously into nude mice (5 mice/each group). GW4064 treatment (30 mg/kg/day) was started when tumor size reaches to 100 mm^3^ (day 0, MCF-7/CAFs group; day 12, MCF-7 group) and delivered daily to the animals by i.p. injection. Relative tumor volume (RTV) was calculated from the following formula: RTV = (Vx/V1), where Vx is the tumor volume on day X and V1 is the tumor volume at initiation of the treatment for each group. Y axis: the mean and ± SD of the RTV. *p < 0.05, GW4064-treated versus vehicle-treated animals. (**b**) Representative Hematoxylin and Eosin (H&E) and **(c)** Azan trichrome stained histologic images of MCF-7/CAF xenograft tumors (20X). (**d**) Human αSMA (*left panel*) and Cytokeratin 18 (*right panel*) immunohistochemical staining of MCF-7/CAF xenograft tumor sections. Scale bars = 25 μm.

**Figure 6:**
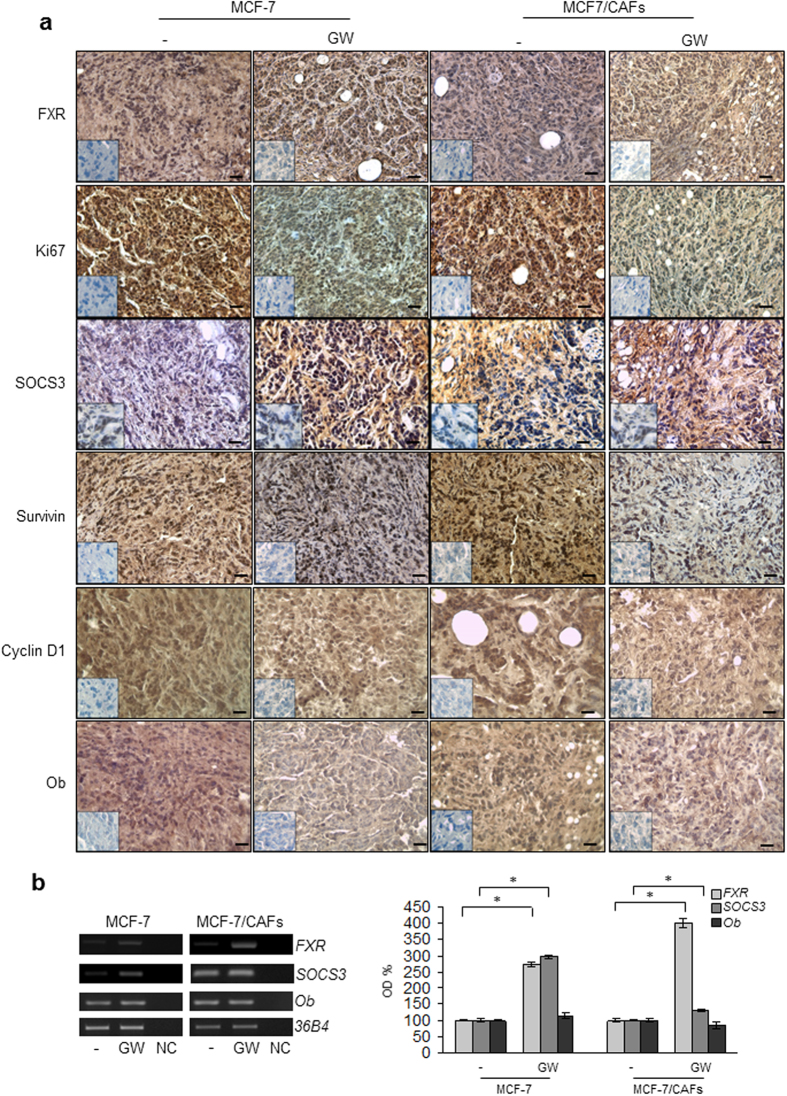
GW4064 decreases leptin target-genes expression in xenograft tumors. (**a**) Representative pictures of FXR, Ki67, SOCS3, Survivin, Cyclin D1 and Ob immunohistochemical staining of MCF-7 and MCF-7/CAF xenograft tumors. Inset: negative control. Scale bars = 25 μm. (**b**) Total RNA from xenografts excised from vehicle and GW4064-treated mice was reverse transcribed and cDNA was subjected to PCR for expression of *FXR*, *SOCS3*, *Ob* and *36B4* (internal standard). NC: negative control. The histograms represent the mean ± SD of separate tumor samples in which band intensities were evaluated in terms of optical density arbitrary units (OD) and expressed as percentage of vehicle-treated samples which were assumed to be 100%. *p < 0.05.

Based on our results demonstrating that FXR activation may counteract tumor-promoting ability of CAFs *in vitro*, we used mouse xenograft models to examine the effect of GW4064 on breast cancer growth *in vivo*. Hence, MCF-7 cells were injected alone or in combination with CAFs into the intrascapular region of female nude mice and tumor growth was monitored upon the administration of vehicle or 30 mg/kg/die GW4064. This treatment was well tolerated because no change in body weight or in food and water consumption was observed, along with no evidence of reduced motor function. In addition, no significant differences in the mean weights or histological features of the major organs (liver, lung, spleen, and kidney) after sacrifice were observed between vehicle-treated mice and those that received treatment, indicating a lack of toxic effects at the dose given. Tumor volume was measured from the first day of treatment and the relative tumor volume was calculated as described in detail in *Materials and Methods*. As shown in Fig. 5a, the co-injection of MCF-7 cells with CAFs determined an impressive acceleration in tumor growth when compared to MCF-7 alone. Importantly, GW4064 treatment induced a significant regression in tumor growth in both MCF-7 and MCF-7/CAF groups. To differentiate epithelial and connective components, MCF-7/CAF samples were stained with hematoxylin and eosin or Azan trichrome stain (Fig. 5b,c, respectively). The same sections were also incubated with anti-human-αSMA and anti-human-Cytokeratin 18 antibodies to verify the human origin of epithelial and connective analyzed tissues (Fig. 5d). Immunostaining of FXR in sections of tumors obtained from GW4064-treated mice revealed an increased immunoreactivity of this receptor, respect to mice treated with vehicle, consistently with the FXR activation by its own ligand (Fig. 6a and Table 1). Moreover, in agreement with our *in vitro* findings, we observed in xenograft tumors from mice treated with GW4064 a significant reduction in the expression of Ki67, a well-known marker for cell proliferation (Fig. 6a and Table 1). Interestingly, in GW4064-treated tumors we showed a strong intensity staining for SOCS3 along with a marked decrease in the expression of Survivin, Cyclin D1, and Ob (Fig. 6a and Table 1). The up-regulation of FXR and SOCS3 was further validated by RT-PCR analysis on tumor tissue samples (Fig. 6b). Moreover, we revealed no significant changes in Ob mRNA levels among vehicle- and GW4064-treated samples (Fig. 6b), suggesting that a different regulation on Ob mRNA and protein levels may occur after GW4064 treatment.

## Discussion

The stromal cells located within the tumor microenvironment are now extensively recognized to directly influence the malignant features of adjacent tumor cells. Interactions between tumor cells and the associated stroma represent a solid relationship that impacts disease initiation, progression and patient prognosis^43^,^44^. Emerging evidences have proposed cancer associated fibroblasts (CAFs) as a pivotal cell type able to shape the architecture of the microenvironment and modulate communications between the various cell types present in the tumor through several signaling molecules, such as growth factors, extracellular matrix-degrading proteases, chemokines and cytokines^14^. Of note, activated stromal fibroblasts have been shown to promote carcinogenesis both *in vitro* and in animal models and an high number of CAFs are often associated with high-grade malignancies and poor prognosis^14^,^45^,^46^. Therefore, a better understanding of the epithelial-stromal interactions has the potential to identify novel therapeutic targets for developing more effective anticancer approaches.

Here, we evidenced, for the first time, that activated FXR, by interfering with the tumor supportive effects of CAFs, is able to inhibit breast cancer growth, motility and invasion. Particularly, we demonstrated that the specific FXR ligand, GW4064, affects the signaling of the adipokine leptin that we have recently identified as one of the most important molecules involved in the tumor-promoting activity of CAFs in breast cancer cells^12^. Our results showed that GW4064 restrains the leptin-induced growth, motility, and invasiveness in both ERα-positive and -negative breast cancer cells. The anti-tumor action of the FXR ligand was associated with the inhibition of several leptin-induced pathways, such as JAK2/STAT3, AKT and MAPK. Interestingly, FXR activation impacts two well-known target genes of leptin, involved in cell proliferation, survival and motility as Cyclin D1, an important regulator of the cell cycle progression, and Survivin, a member of the inhibitor of apoptosis protein family^33^,^36^,^38^,^47^,^48^. Moreover, the FXR ligand induces a decrease on leptin-mediated up-regulation of its own gene (*Ob*) expression, highlighting how this nuclear receptor is able to negatively interfere in the short autocrine loop maintained by leptin on *Ob* gene in breast cancer cells. The above described effects were strictly dependent on FXR activation since they disappear in the presence of the dominant negative FXR plasmid. Mechanistically, we discovered that activated FXR counteracts leptin action on breast cancer cells through its ability to induce the expression of the suppressor of cytokine signaling 3 (SOCS3), a prototype molecule of the SOCS family. SOCS proteins are negative regulators of the JAK/STAT pathway and inhibit cytokine signaling by preventing JAK activity or by promoting protein degradation^39^,^49^,^50^,^51^. SOCS3 has been identified as an inducible suppressor of leptin signaling, and in breast cancer cells, its overexpression has been shown to decrease proliferation and anchorage-independent growth^48^. Recently, SOCS3 was recognized as a direct FXR target gene. Indeed, it has been demonstrated, in hepatocytes, that FXR ligands upregulate SOCS3 expression by enhancing its promoter activity^41^. Besides, FXR^−/−^ knockout mice exhibit reduced basal expression of SOCS3, whereas activation of FXR by GW4064 induces SOCS3 mRNA levels in wild-type but not in FXR^−/−^ mice^42^. In line with these observations, our study demonstrated a direct involvement of FXR in positively modulating SOCS3 expression as revealed by its increase at both mRNA and protein levels in cells treated with GW4064. Furthermore, data from knock-down experiments highlight the functional relevance of SOCS3 in mediating the inhibitory effects of activated FXR on leptin downstream signaling.

During breast carcinogenesis, the epithelial and stromal compartments coevolve not only through cell-cell contact but also by secretion of soluble effectors coming from surrounding stromal cells. Indeed, in breast cancer tissues, ductal carcinoma *in situ* (DCIS) exhibits, in the stroma compartment, changes in the gene expression in a greater extent respect to the invasive tumors^52^. Since the basement membrane is largely intact in DCIS, this suggests that paracrine and endocrine effects are crucial in inducing these modifications. It is now well accepted that the tumor along with its surrounding stromal tissue promotes a leptin-rich environment which contributes to tumor development and progression^1^,^2^,^12^. In this context, we demonstrated that CAFs, the principal cellular component of the stroma, express leptin receptor and secrete leptin, which sustains a short autocrine loop and is able to target tumor epithelial cells enhancing breast cancer cell growth and invasiveness^12^. Our results show that activated FXR may be able to counteract the leptin-dependent paracrine effects on breast cancer restraining the tumor-promoting activities exerted by CAFs.

The physiological relevance of the inhibitory effects exerted by FXR ligand on breast tumor cells is pointed out by our *in vivo* studies showing that GW4064 administration markedly reduced growth of both MCF-7 as well as MCF-7/CAF xenografts. These observations well correlated with previous reports from our research group demonstrating that FXR activation plays a crucial role in reducing breast cancer cell proliferation^28^ and in inhibiting testicular tumor growth *in vitro* and *in vivo*^25^,^26^. Of note, we observed in tumor sections from GW4064-treated mice a significant decrease in the expression of leptin-regulated proteins and this was concomitant with a strong positivity for SOCS3, suggesting that an inhibition of leptin signaling may be involved in the reduction of tumor growth *in vivo* induced by activated FXR on both MCF-7 alone and MCF-7 co-injected with CAFs.

Although the therapeutic strategies against breast cancer have been focused for long time on directly targeting the malignant cell itself, it is now well recognized that the specific control of the signaling coming from the surrounding stromal cells represents an even more compelling treatment option. Thus, our results, highlighting the ability of activated FXR to counteract leptin signaling responsible for mammary carcinogenesis within the tumor microenvironment, support the possibility that FXR ligands could represent a promising pharmacological tools to be exploited in the novel strategies for breast cancer treatment.

## Materials and Methods

### Reagents and antibodies

GW4064 from Tocris Bioscience (Bristol, UK). L-glutamine, penicillin, streptomycin, aprotinin, leupeptin, phenylmethylsulfonyl fluoride (PMSF), sodium orthovanadate, NP-40, MTT and anti-αSMA antibody were purchased from Sigma (Milan, Italy). Dulbecco’s Modified Eagle’s Medium (DMEM), DMEM-F12, RPMI, McCoy’s5A, Hank’s balanced salt solution, fetal bovine serum (FBS), Leptin, TRIzol, TaqDNA polymerase, RETROscript kit, 100-bp DNA ladder were from Life Technologies (Monza MB, Italy). Antibodies against FXR, Survivin, Cyclin D1, Ob, KI67, Cytokeratin 18, and β-Actin, by Santa Cruz Biotechnology (Santa Cruz, CA, USA); anti-SOCS3 antibody by Abcam (Cambridge, UK). Antibodies against total non-phosphorylated and Phosphorylated (p) JAK2 (Tyr^1007/1008^), STAT3 (Tyr^705^), Akt (Ser^473^), and MAPK (Thr^202^/Tyr^204^) were purchased from Cell Signaling Technology (Beverly, MA, USA).

### Cell culture

MCF-7, MDA-MB-231 and SKBR3 human breast cancer cell lines were acquired from American Type Culture Collection (Manassas, VA, USA) where they were authenticated, stored according to supplier’s instructions, and used within 4–6 months after frozen aliquots resuscitations.

MCF-7 cells were cultured in DMEM medium containing 10% FBS, 1% L-glutamine, 1% Eagle’s nonessential amino acids, and 1 mg/ml penicillin-streptomycin at 37 °C with 5% CO_2_ air. MDA-MB-231 cells were grown in DMEM:F12 containing 10% FBS. SKBR3 cells were cultured in McCoy’s5A Medium modified containing 10% FBS. Before each experiment, cells were grown in phenol red-free medium, containing 5% charcoal-stripped FBS (cs-FBS) for 2 days and treated as described.

### CAF isolation

Human breast cancer specimens were collected in 2013–2014 from four primary tumors of patients who signed informed consent in accordance with approved Human Subject’s guidelines at Annunziata Hospital (Cosenza, Italy). Following tumor excision, small pieces were digested (500 IU collagenase in Hank’s balanced salt solution; Sigma; 37 °C for 2 h). After differential centrifugation (90 × g for 2 min), the supernatant containing CAFs was centrifuged (500 × g for 8 min), resuspended, and cultured in RPMI-1640 medium supplemented with 15% FBS and antibiotics. CAFs between 4 and 10 passages were used, tested by mycoplasma presence (MycoAlert Mycoplasma Detection Assay, Lonza), and authenticated by αSMA and fibroblast activation protein (FAP) expression. Study was approved by ethics institutional committees at Annunziata Hospital. Experimental procedures for CAF isolation were carried out in accordance with the approved guidelines.

### Leptin measurement by ELISA

Leptin was measured by ELISA (LDN, Nordhorn, Germany) following manufacturer’s protocol. Results are presented as nanograms per mg of protein.

### Immunofluorescence

Cells were fixed with 4% paraformaldehyde, permeabilized with PBS 0.2% Triton X-100 followed by blocking with 5% bovine serum albumin, and incubated with anti-human-αSMA antibody and with fluorescein isothiocyanate-conjugated secondary antibody. IgG primary antibody was used as negative control. 4′,6-Diamidino-2-phenylindole (DAPI; Sigma) staining was used for nuclei detection. Fluorescence was photographed with OLYMPUS BX51 microscope, 20x objective.

### Conditioned medium systems

CAFs were incubated with 5% cs-FBS- phenol-red free media (48–72 h). Conditioned media (CAF-CM) were collected, centrifuged to remove cellular debris, and used in respective experiments.

### Soft agar growth assays

Cells (25,000 per well) were plated in 4 ml of 0.35% agarose with 5% CS-FBS in phenol red-free media, in a 0.7% agarose base in six-well plates. Two days after plating, media containing control vehicle or treatments was added to the top layer, and the media were replaced every two days. After 14 days, 150 μl of MTT was added to each well and allowed to incubate at 37 °C for 4 h. Plates were then placed in 4 °C overnight and colonies >50 μm diameter from triplicate assays were counted.

### Wound-healing assays

Cell monolayers were scraped and treated as indicated. Wound closure was monitored over 12–24 h; cells were fixed and stained with Coomassie brilliant blue. Pictures were taken at 10X magnification using phase-contrast microscopy and are representative of three independent experiments. The rate of wound healing was quantified from the images using Scion Image Program and Adobe Photoshop Program and standard deviations along with associated P values for the biological replicates were determined by using GraphPad-Prism4 software (GraphPad Inc., San Diego, CA).

### Invasion assays

Matrigel-based invasion assay was conducted in invasion chambers (8-μm membranes) coated with Matrigel (BD Biosciences; 0.4 μg/mL). Cells treated with leptin, CAF-CM with or without GW4064 were seeded into top Transwell chambers, whereas regular full medium was used as chemoattractant in lower chambers. After 24 h (MDA-MB-231) and 36 h (MCF-7), invaded cells were fixed with 4% paraformaldehyde and stained with DAPI. Images represent 1 of 3 independent experiments (10X magnification).

### Immunoblot analysis

MCF-7, MDA-MB-231 and SKBR3 cells were grown to 50–60% confluence and treated as indicated before lysis in 500 μl of 50 mM Tris-HCl, 150 mM NaCl, 1% NP-40, 0.5% sodium deoxycholate, 2 mM sodium fluoride, 2 mM EDTA, 0.1% SDS, containing a mixture of protease inhibitors (aprotinin, phenylmethylsulfonyl fluoride, and sodium orthovanadate) for total protein extraction. Equal amounts of proteins were resolved on 11% SDS-polyacrylamide gel, transferred to a nitrocellulose membrane and probed with specific antibodies as described. To ensure equal loading, all membranes were stripped and incubated with anti-β-Actin antibody. The antigen-antibody complex was detected by incubation of the membranes with peroxidase-coupled goat anti-mouse or goat anti-rabbit antibodies and revealed using the ECL System. Blots are representative of three independent experiments. The bands of interest were quantified by Scion Image laser densitometry scanning program and standard deviations along with associated P values for the biological replicates were determined by using GraphPad-Prism4 software.

### Reverse transcription–PCR assays

Total RNA was extracted from MCF-7, MDA-MB-231, SKBR3 and CAF cells and from xenografts tissues using TRIzol reagent and the evaluation of FXR, SOCS3, Ob, ObR, FAP and 36B4 gene expression was performed by the reverse transcription-PCR method using a RETROscript kit. The cDNAs obtained were amplified by PCR using the primers listed in Supplementary Table S1 online. Negative control contained water instead of first strand cDNA.

### Transient transfection assays

MCF-7 cells were transiently transfected using the FuGENE 6 reagent with either empty vector (e.v.) or FXR-DN plasmid for 24 h and then cells were treated as described. The FXR-DN expression plasmids were provided from Dr T.A. Kocarek (Institute of Environmental Health Sciences, Wayne State University, USA)^53^.

### RNA silencing

MCF-7 cells were transiently transfected with a siRNA targeted for the human SOCS3 RNA sequence (Qiagen, S103060358), or with a control siRNA (Qiagen, S100300650) to a final concentration of 30 nM using Lipofectamine 2000 as recommended by the manufacturer. After 24 h of transfection, cells were exposed to treatments.

### *In vivo* experiments

The *in vivo* experiments were done in 45-day-old female athymic nude mice (Harlan Laboratories, Udine, Italy) maintained in a sterile environment. The animals were fully anesthetized by i.p. injection of chloral hydrate 400 mg/kg to allow the s.c. implantation of estradiol (E_2_) pellets (1.7 mg per pellet, 60-day release; Innovative Research of America, Sarasota, FL) into the intrascapular region. The day after, exponentially growing MCF-7 cells (5.0 × 10^6^ cells per mouse) alone or in combination with CAFs were inoculated s.c. in 0.1 mL of Matrigel. In the group receiving both tumor cells and CAFs, a ratio 3:1 (tumor cells/CAFs) was used. The subcutaneous location was used to eliminate the influence of other mammary gland cellular components on the interaction of human breast fibroblasts and epithelial cells. GW4064 treatment (30 mg/kg/day) was started when mean tumor size reaches to 100 mm^3^ (day 0) and delivered daily to the animals by i.p. injection. Tumor development was followed twice a week by calliper measurements along two orthogonal axes: length (L) and width (W). The volume (V) of tumors was estimated by the following formula: V = L X (W^2^)/2. Relative tumor volume (RTV) was calculated from the following formula: RTV = (Vx/V1), where Vx is the tumor volume on day X and V1 is the tumor volume at initiation of the treatment. Growth curve was obtained by plotting the mean volume of RTV on Y axis against time (X axis expressed as days after starting of treatment). At day 32 the animals were sacrificed following the standard protocols and tumors were dissected from the neighboring connective tissue. Specimens of tumors were frozen in nitrogen and stored at −80 °C. The remaining tumor tissues of each sample were fixed in 4% paraformaldehyde and embedded in paraffin for the histological analyses. All animals were maintained and handled in accordance with the recommendation of the Guidelines for the Care and Use of Laboratory Animals and were approved by the Animal Care Committee of University of Calabria, Italy.

### Histopathological analysis

Tumors, livers, lungs, spleens and kidneys were fixed in 4% formalin, sectioned at 5 μm and stained with haematoxylin and eosin Y, as suggested by the manufacturer (Bio-Optica, Milan, Italy). Some sections from MCF-7/CAFs tumors were stained with Azan trichrome (Bio-Optica, Milan, Italy) to differentiate epithelial and connective components.

### Immunohistochemical analysis

Paraffin-embedded sections, 5 μm thick, were mounted on slides precoated with poly-lysine, and then they were deparaffinised and dehydrated (seven to eight serial sections). Immunohistochemical experiments were performed after heat-mediated antigen retrieval, using human Cytocheratin 18, αSMA primary antibodies in MCF-7/CAF samples and FXR, Ki67, SOCS3, Ob, Survivin and Cyclin D1 primary antibodies in both MCF-7 and MCF-7/CAF samples at 4 °C overnight. Then, a biotinylated specific IgG was applied for 1 h at room temperature, followed by the avidin biotin horseradish peroxidase complex (Vector Laboratories, CA). Immunoreactivity was visualized by using the diaminobenzidine chromogen (Sigma-Aldrich). Counterstaining was carried out with haematoxylin (Sigma-Aldrich). The primary antibody was replaced by normal serum in negative control sections. The immunostained slides of tumour samples were evaluated by light microscopy using the Allred Score^54^, which combines a proportion score and an intensity score. A proportion score was assigned representing the estimated proportion of positively stained tumor cells (0 = none; 1 = 1/100; 2 = 1/100 to < 1/10; 3 = 1/10 to <1/3; 4 = 1/3 to 2/3; 5 = > 2/3). An intensity score was assigned by the average estimated intensity of staining in positive cells (0 = none; 1 = weak; 2 = moderate; 3 = strong). Proportion score and intensity score were added to obtain a total score that ranged from 0 to 8. A minimum of 100 cells were evaluated in each slide. Six to seven serial sections were scored in a blinded manner for each sample.

### Statistical analyses

Each datum point represents the mean ± S.D. of three different experiments. Data were analyzed by Student’s t test using the GraphPad Prism 4 software program. For immunohistochemistry, the differences in the scores between GW-treated and vehicle-treated samples were examined by one-way ANOVA. *P < 0.05 was considered as statistically significant.

## Additional Information

**How to cite this article**: Giordano, C. *et al.* Activated FXR Inhibits Leptin Signaling and Counteracts Tumor-promoting Activities of Cancer-Associated Fibroblasts in Breast Malignancy. *Sci. Rep.* **6**, 21782; doi: 10.1038/srep21782 (2016).

## Supplementary Material

Supplementary Information

## Figures and Tables

**Table 1:** Immunohistochemistry scores in MCF-7 and MCF-7/CAF xenograft tumors.

Antibody	IHC score in tumor cells			
	MCF-7		MCF-7/CAFs	
	–	GW4064	–	GW4064
FXR	4	6*	3	6*
Ki67	8	2*	8	2*
SOCS3	1	5*	2	6*
Survivin	5	2*	6	1*
Cyclin D1	6	5*	7	4*
Ob	5	3*	4	2*

Note: Cases were scored according to Allred immunohistochemistry (IHC) score^54^ which includes both the proportion and intensity scores (range, from 0 to 8). *P < 0.001 GW4064-treated versus vehicle-treated samples. FXR: Farnesoid X Receptor; SOCS3: Suppressor of cytokine signaling 3; Ob: Leptin.
