# Supplementary material for: Activated FXR Inhibits Leptin Signaling and Counteracts Tumor-promoting Activities of Cancer-Associated Fibroblasts in Breast Malignancy
Source: Sci Rep. 2016 Feb 22;6:21782. doi: 10.1038/srep21782 (PMC4761870; doi:10.1038/srep21782)
Supplement: Supplementary Information [file srep21782-s1.pdf]

Activated FXR Inhibits Leptin Signaling and Counteracts Tumor-promoting Activities of Cancer-Associated Fibroblasts in Breast Malignancy.

Cinzia Giordano[1,#], Ines Barone[2,#], Valentina Vircillo[2], Salvatore Panza[2], Rocco Malivindi[2], Luca Gelsomino[2], Michele Pellegrino[2], Vittoria Rago[2], Loredana Mauro[2], Marilena Lanzino[2], Maria Luisa Panno[2], Daniela Bonofiglio[2], Stefania Catalano[2,*], Sebastiano Andò[1,2,*]

Author Affiliations:
[1]Centro Sanitario, [2]Department of Pharmacy, Health and Nutritional Sciences, University of Calabria, Arcavacata di Rende, CS, Italy;

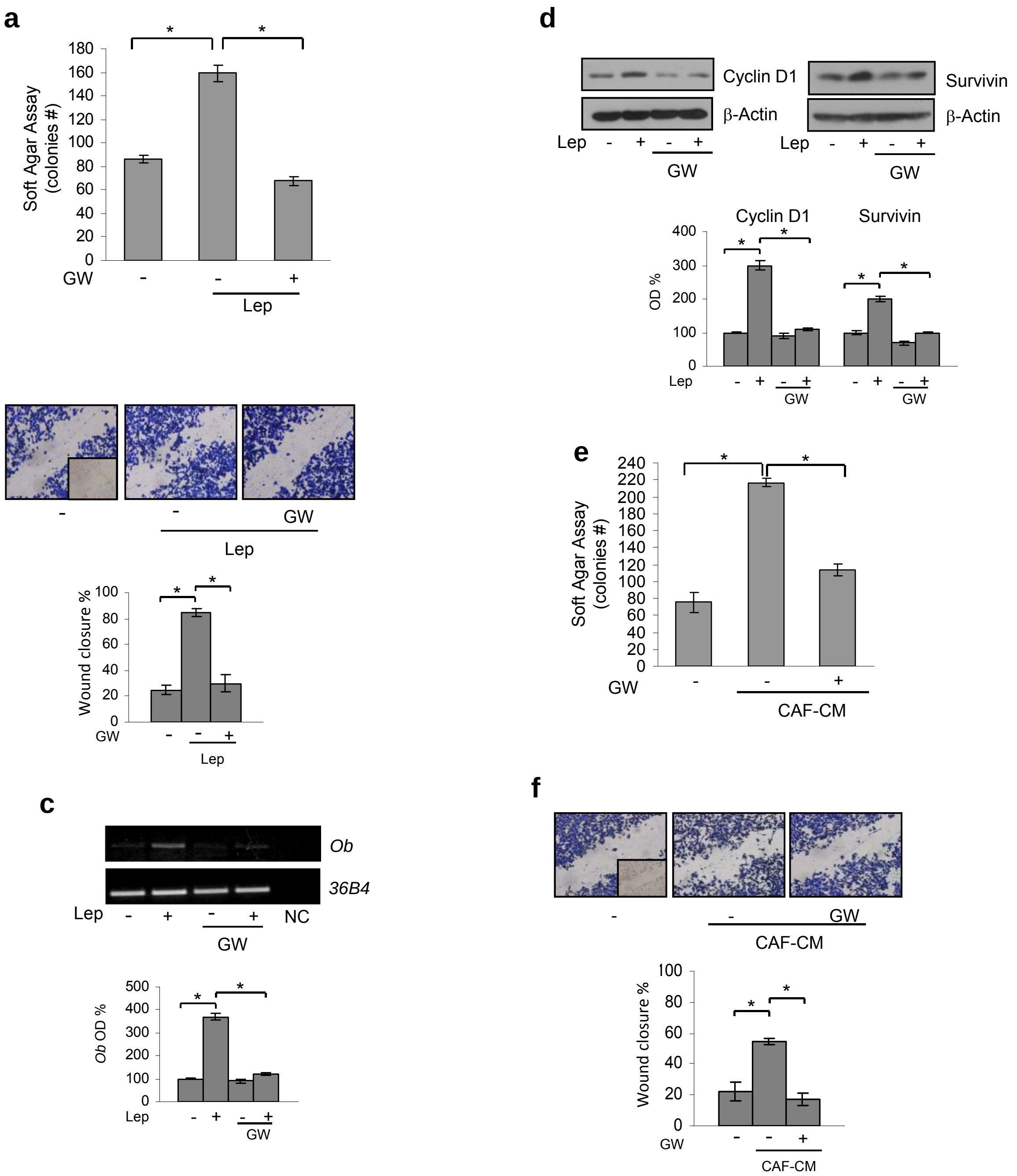

Supplementary Figure S1. Effects of activated FXR in SKBR3 breast cancer cells. (a) Soft Agar growth assay in SKBR3 cells treated with Leptin (Lep, 500ng/ml) with/without GW4064 (6μM). **(b)** Wound-healing assay in cells treated as indicated for 24h. *Upper panel*, representative images from each condition (inset, time 0). *Lower panel*, relative percentage of wound closure. **(c)** RT-PCR analysis of leptin (*Ob*) mRNA levels. *36B4,* internal standard. NC, negative control. **(d)** Immunoblot analysis for Survivin, Cyclin D1, β-Actin (loading control) expression. *Lower panels* for (**c**) and (**d**), the histograms represent the mean ± SD of three separate experiments in which band intensities were evaluated in terms of optical density arbitrary units (OD), expressed as percentage of vehicle-treated samples assumed to be 100%. (**e**) Soft Agar growth assay in cells treated with CAF-CM with/without GW4064. **(f)** Wound-healing assay in cells treated as indicated. *Upper panel*, representative images from each condition (inset, time 0). *Lower panel*, relative percentage of wound closure. The values represent the mean ± SD of three independent experiments. $^*p < 0.05$.

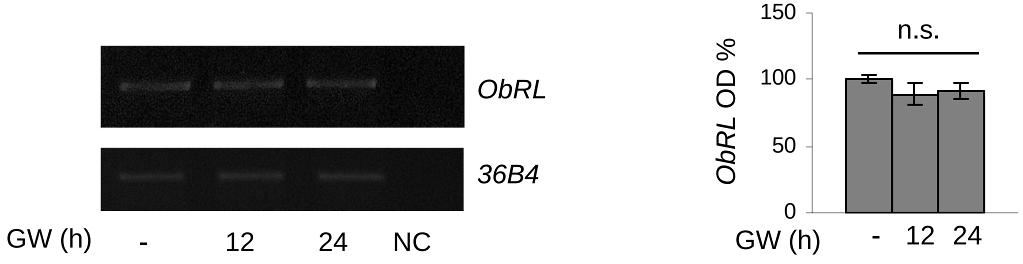

Supplementary Figure S2. Effects of activated FXR on leptin receptor expression in MCF-7 breast cancer cells. Leptin receptor long isoform (*ObRL*) mRNA expression in MCF-7 cells treated for 12 or 24 hours with GW4064 (GW, 6μM) by RT-PCR analysis. *36B4* (internal standard). NC, negative control. The histograms represent the mean ±SD of three separate experiments in which band intensities were evaluated in terms of optical density arbitrary units (OD) and expressed as percentage of vehicle-treated samples which were assumed to be 100%; n.s: nonsignificant.

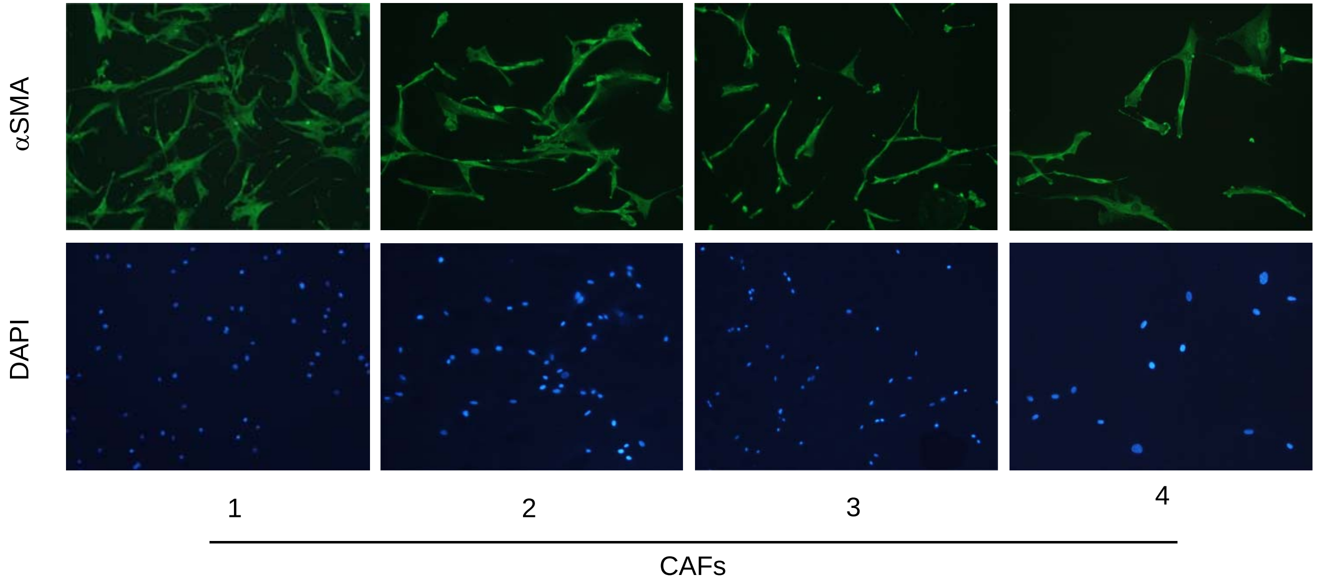

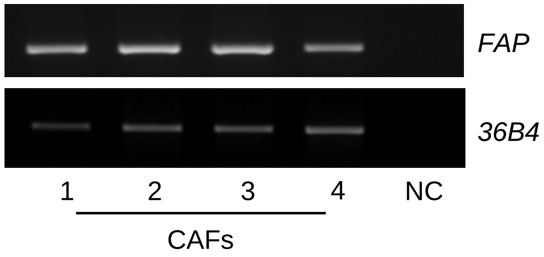

Supplementary Figure S3. Characterization of Cancer-Associated Fibroblasts. Cancer-Associated Fibroblasts (CAFs) were isolated from four primary breast tumour biopsies by collagenase digestion. *Upper panel*, immunofluorescence of alpha-smooth muscle actin (α-SMA) and DAPI staining (for nuclei detection); *lower panel*, RT-PCR analysis of Fibroblast Activation Protein (*FAP*) mRNA levels. *36B4* (internal standard). NC, negative control.

Supplementary Table S1. Oligonucleotide primers used in this study.

Gene Symbol	Gene Name	Primer Sequences
SOCS3	Suppressor of cytokine signaling 3	For 5’-ATCCTGGTGACATGCTCCTC- 3’<br>Rev 5’- GGCACCAGGTAGACTTTGGA-3’
Ob	Leptin	For 5’ - CTGTGCCCATCCAAAAAGTCC-3’<br>Rev 5’- CCCCCAGGCTGTCCAAGGTC-3’
ObRL	Leptin Receptor Long Isoform	For 5’-CGAGAAACGTTTCAGCATCT-3’<br>Rev 5’- CAAAAGCACACCACTCTCTC-3’
FAP	Fibroblast activation protein	For 5’-AGAAAGCAGAACTGGATGG-3’<br>Rev 5’-ACACACTTCTTGCTTGGAGGAT-3’
FXR	Nuclear receptor subfamily 1, group H, member 4 (NR1H4)	For 5’-CGAGCCTGAAGAGTGGTACTGTC-3’<br>Rev 5’- CATTCAGCCAACATTCCCATCTC-3’
36B4	36B4	For 5’-CTCAACATCTCCCCCTTCTC-3’<br>Rev 5’- CAAATCCCATATCCTCGT -3’
